# Physalin F, a Potent Inhibitor of Lymphocyte Function, Is a Calcineurin Inhibitor and Has Synergistic Effect with Dexamethasone

**DOI:** 10.3390/molecules30040916

**Published:** 2025-02-16

**Authors:** Dahara Keyse Carvalho Silva, Laura Beatriz da Cruz Novo, Ivone Maria Ribeiro, Breno Cardim Barreto, Luiza Carolina França Opretzka, Cássio Santana Meira, Milena Botelho Pereira Soares

**Affiliations:** 1 Gonçalo Moniz Institute, Oswaldo Cruz Foundation (FIOCRUZ), Salvador 40296-710, BA, Brazil; daharacarvalho@gmail.com (D.K.C.S.); lauranovo@ufba.br (L.B.d.C.N.); cassio.meira@fieb.org.br (C.S.M.); 2 Laboratory of Natural Products Chemistry—PN2, Farmanguinhos, Oswaldo Cruz Foundation (FIOCRUZ), Rio de Janeiro 22775-903, RJ, Brazil; ribeiroivone15@yahoo.com.br; 3 Institute for Innovation in Advanced Health Systems, National Service for Industrial Learning—Integrated Center for Manufacturing and Technology (SENAI CIMATEC), Salvador 41650-010, BA, Brazil; brenoc.barreto@hotmail.com (B.C.B.); luizacfo@gmail.com (L.C.F.O.)

**Keywords:** physalin F, lymphocytes, immunomodulation, combination therapy

## Abstract

The dysregulation of immune responses are responsible for the development of several diseases, such as allergic and autoimmune diseases. The medications used to treat these conditions have numerous side effects, creating the need for new drugs. Physalins are natural compounds with various pharmacological activities already described. Here, we aimed to investigate the immunomodulatory effects of physalin F in mouse splenocytes and in a delayed-type hypersensitivity (DTH) model. In a cytotoxicity assay, physalin F had low cytotoxicity to mouse splenocytes in concentrations equal to or below 2 µM. It significantly inhibited lymphocyte proliferation in a concentration-dependent manner and reduced the production of cytokines, including IL-2, IL-4, IL-10, and IFN-γ, in activated splenocytes. The combined therapy of physalin F with dexamethasone was investigated in vitro, showing a synergistic action of the two compounds. Mechanistically, physalin F reduced calcineurin activity in concanavalin A-stimulated splenocyte cultures. Finally, in vivo, the intraperitoneal administration of physalin F in a DTH model reduced paw edema induced by bovine serum albumin immunization. Our results demonstrate the potential of physalin F as an immunosuppressive agent, to be used alone or in combination with glucocorticoids.

## 1. Introduction

The immune system plays a crucial role in protecting the body against a wide range of pathogens and facilitating the resolution of tissue injuries [1,2]. However, during the inflammatory process, the excessive production of pro-inflammatory mediators, such as nitric oxide (NO), tumor necrosis factor-alpha (TNF-α), interleukins, chemokines, adhesion molecules, and eicosanoids, can lead to significant tissue damage [3]. This dysregulated response is associated with the development of various inflammatory diseases, autoimmune disorders, and allergies, particularly when the immune system is activated in an undesirable or exaggerated manner. Dysfunctions in the activity of lymphocytes and macrophages are key factors driving the onset and progression of these conditions [4]. Lymphocytes, in particular, play a central role in immune recognition, serving as essential cellular components in the orchestration of the immune response.

To treat these inflammatory diseases, the most recommended medications are nonsteroidal anti-inflammatory drugs and corticosteroids. However, the use of these medications is associated with various adverse effects [5,6]. For instance, the long-term use of corticosteroids is linked to a range of adverse effects, requiring constant monitoring of the patient. Since they alter the body’s general metabolism, corticosteroids can reduce glucose uptake and utilization, increase gluconeogenesis, promote rebound hyperglycemia and glycosuria, and increase catabolism, while reducing protein anabolism. The main adverse effects associated with their use include diabetes, susceptibility to infections, osteoporosis, cataracts, hypertension, myopathies, neurological disorders, nephrotoxicity, and an increased risk for the development of cardiovascular diseases [7].

Therefore, there is a need for new therapeutic alternatives, especially to replace corticosteroids. In this context, natural compounds represent a promising source for the development of new therapeutic agents [8,9]. The use of medicinal plants to treat various diseases is a practice that has been recognized and utilized for thousands of years. Currently, more than 50% of available drugs are derived from or inspired by natural products [9].

Physalins, or 16,24-cyclo-13,14-seco steroids, are compounds found in plants belonging to the Solanaceae family, mainly in the species of the genus *Physalis* spp. [10]. These compounds have a wide range of pharmacological activities, including anti-inflammatory, antitumor, antimicrobial, antinociceptive, antiviral, and antiparasitic properties [11,12,13,14,15,16,17,18]. Over 75 physalins are known, with physalins A, B, D, F, G, and H being the most studied. Among them, physalin F stands out for its potent immunomodulatory activity [14,18,19]. However, there is a need to better understand its effects on lymphocyte function. In this context, the present study aims to investigate the immunomodulatory effects of physalin F in mouse lymphocytes and, in vivo, in a model of delayed-type hypersensitivity response.

## 2. Results and Discussion

**Figure 1:**
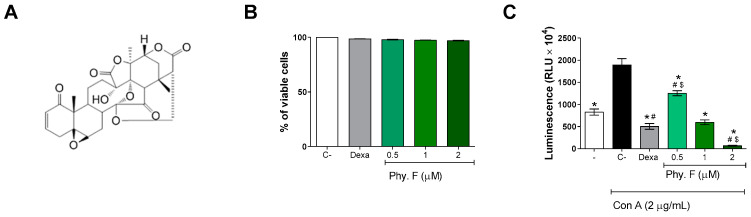
Physalin F inhibits lymphoproliferation at non-cytotoxic concentrations. (**A**) Chemical structure of physalin F. (**B**) The cytotoxicity of physalin F was assessed after 72 h incubation in splenocyte cultures of BALB/c mice, using propidium iodide staining, followed by acquisition and analysis by flow cytometry. (**C**) Lymphoproliferation was assessed in splenocyte cultures from BALB/c mice, stimulated or not with concanavalin A (Con A; 2 µg/mL) and treated or not with physalin F (Phy. F; 0.5, 1, or 2 µM) or dexamethasone (Dexa; 1 µM) for 72 h, using a luminescent assay. - is unstimulated and untreated cells. C- is stimulated and untreated cells. Values are expressed as mean ± S.E.M. of four determinations, representing one of three independent experiments. Statistical significance was determined as * *p* < 0.05 compared to stimulated, untreated cells. # *p* < 0.05 compared to unstimulated, untreated cells. $ *p* < 0.05 compared to stimulated cells treated with dexamethasone.

First, the cytotoxicity of physalin F (Figure 1A) was evaluated in splenocyte cultures incubated with physalin F for 72 h, after staining with propidium iodide (PI) and analysis with flow cytometry. The percentage of PI-positive cells was below 5% at 0.5, 1, and 2 µM of physalin F, indicating a low cytotoxicity of this compound at the concentrations tested. Previous studies also assessed the cytotoxicity of physalin F at higher concentrations in splenocytes, reporting an LC_50_ value of 13.3 µM [20]. These findings further corroborate the low cytotoxicity observed in the present study. Similarly, dexamethasone demonstrated low cytotoxicity at a concentration of 1 µM after 72 h of treatment (Figure 1B and Figure S1).

Next, the effect of physalin F on mouse splenocytes stimulated with concanavalin A (Con A) was evaluated. At all tested concentrations, physalin F inhibited lymphoproliferation in a concentration-dependent manner, 72 h after treatment (Figure 1C). At 2 µM, physalin F inhibited about 95% compared to untreated Con A-stimulated cells. At 1 µM, physalin F inhibited approximately 68% of lymphoproliferation, while the lowest concentration (0.5 µM) achieved an inhibition of approximately 33%. As expected, dexamethasone (1 µM) also inhibited lymphoproliferation (Figure 1C). The results are consistent with the literature, where physalins B, F, G, and H have previously demonstrated their antiproliferative effects in activated splenocyte cultures using ^3^H-thymidine incorporation assay [21,22]. Among the physalins evaluated in lymphoproliferation assays, physalin F stands out as the most active and promising molecule for this activity [21]. Notably, physalin F also exhibits a concentration-dependent inhibition of spontaneous proliferation in PBMCs derived from individuals with human T-lymphotropic virus type 1 (HTLV-1)-associated with myelopathy/tropical spastic paraparesis (HAM/TSP) [19].

**Figure 2:**
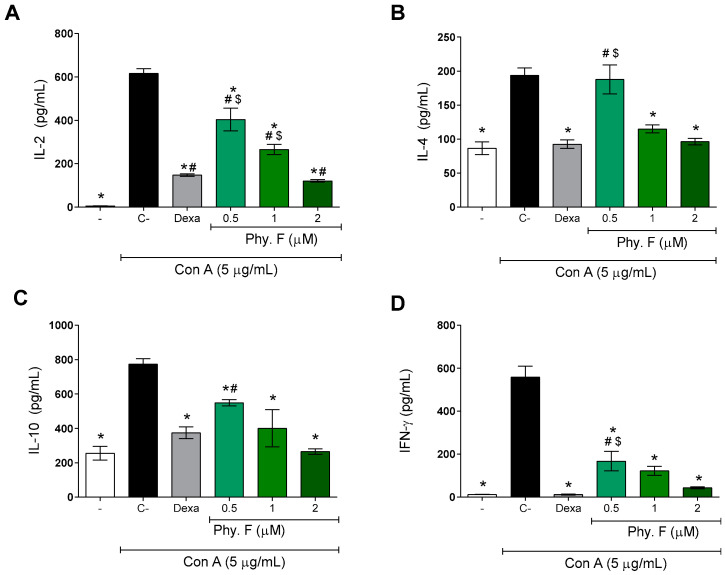
Physalin F reduces cytokine production by mitogen-activated lymphocytes. The concentrations of cytokines IL-2 (**A**), IL-4 (**B**), IL-10 (**C**), and IFN-γ (**D**) were measured in supernatants of splenocyte cultures stimulated with concanavalin A (Con A; 5 µg/mL) and treated or not with physalin F (Phy. F; 0.5, 1, or 2 µM) or dexamethasone (Dexa; 1 µM) for 48 h. Cytokine quantification was performed by ELISA. - is unstimulated and untreated cells. C- is stimulated and untreated cells. Values are expressed as mean ± S.E.M. of six determinations, representing one of three independent experiments. Statistical significance was determined as * *p* < 0.05 compared to stimulated, untreated cells. # *p* < 0.05 compared to unstimulated, untreated cells. $ *p* < 0.05 compared to stimulated cells treated with dexamethasone.

Subsequently, the effect of physalin F was evaluated on the production of IL-2, IL-4, IL-10, and IFN-γ. After 48 h of treatment with physalin F, the levels of Th1 (IL-2 and IFNγ) and Th2 (IL-4 and IL-10) cytokines were significantly reduced by physalin F treatment (*p* < 0.05) (Figure 2). Under the same conditions, dexamethasone (1 µM) also promoted a statistically significant (*p* < 0.05) reduction in the four cytokines evaluated (Figure 2). Importantly, the effects of physalin F at 2 µM were equal to or superior to that observed with dexamethasone, a gold-standard immunosuppressive agent.

The inhibition promoted by physalin F on the production of inflammatory mediators highlights its potent immunosuppressive action. IL-2 is one of the main cytokines involved in lymphocyte activation and proliferation, playing a critical role in the induction and maintenance of regulatory T cells [23]. IFN-γ, while essential for immunological competence, can cause excessive tissue damage, necrosis, and inflammation when overactive, contributing to the pathology of various diseases [24]. IL-10 is produced by lymphocytes, particularly by Th2 and Tregs, and plays a crucial role in maintaining immune homeostasis. It suppresses the production of pro-inflammatory cytokines, limits the activation of effector T cells, and promotes the resolution of inflammation, thereby preventing excessive immune responses and tissue damage [25]. Similarly, IL-4 is crucial for the differentiation of T cells into the Th2 profile and humoral responses. However, IL-4 also plays a role in the persistence of asthma and other allergic diseases [26,27,28].

A previous study has shown that physalin H, similar to physalin F, is able to inhibit the production of inflammatory mediators, such as IL-2 and IFNγ, in T lymphocyte cultures from BALB/c mice [22]. However, unlike what was observed with physalin F, physalin H increased the production of IL-4 and IL-10 [22]. Moreover, physalin F also inhibited the production of IL-2, IL-10, and IFNγ by PBMCs obtained from patients infected with HTLV-1 [19].

**Figure 3:**
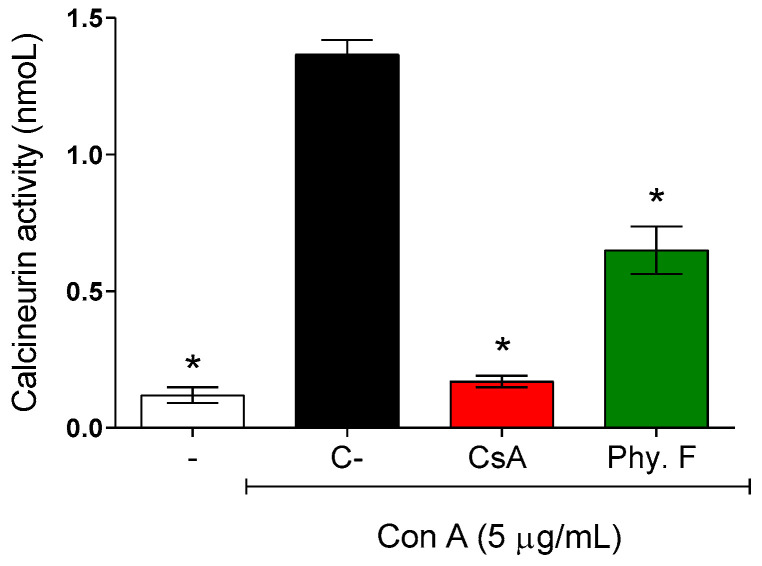
Evaluation of calcineurin activity in activated lymphocytes treated or not with physalin F. Mouse splenocytes were stimulated with concanavalin A (Con A; 5 µg/mL) and treated or not with physalin F (Phy. F; 1 µM) or cyclosporine A (CsA; 1 µM) for 48 h. The activity of calcineurin was assessed in the cell lysate, using a colorimetric assay. - is unstimulated and untreated cells. C- is stimulated and untreated cells. Values are expressed as mean ± S.E.M. of three determinations, representing one of three independent experiments. Statistical significance was determined as * *p* < 0.05 compared to Con A stimulated, untreated cells.

To better understand the mechanisms of action of physalin F underlying its immunosuppressive effects on splenocytes, its effect on calcineurin activity was evaluated. Mouse splenocytes were activated with concanavalin A (5 µg/mL) in the absence or presence of physalin F. Treatment with physalin F (1 µM) for 48 h resulted in a 52.3% reduction in calcineurin activity compared to the control untreated. Under the same conditions, cyclosporine A, a standard inhibitor of calcineurin, reduced calcineurin activity by 87.6% (Figure 3).

The mechanism underlying the immunomodulatory activity of physalin F remains unknown. A study by Jacobo-Herrera and colleagues [29] suggested that this compound may exert an effect on NF-κB activity. Here, we showed, for the first time, the potential action of physalin F on calcineurin activity. Calcineurin is a phosphatase complex that plays a pivotal role in several cellular processes and calcium-dependent signal transduction pathways, including T cell activation [30]. Classic calcineurin inhibitor immunosuppressants, such as cyclosporine and tacrolimus, exemplify the clinical relevance of targeting this pathway for the treatment of immune-mediated diseases [31].

**Figure 4:**
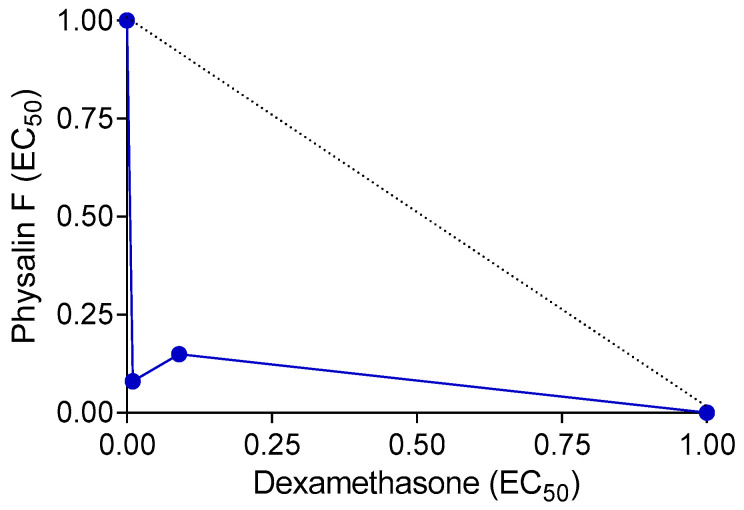
Isobologram depicting the synergistic effects of physalin F and dexamethasone on lymphocyte proliferation. Lymphoproliferation was assessed in splenocyte cultures from BALB/c mice, stimulated or unstimulated with concanavalin A, and treated with physalin F and/or dexamethasone at varying concentrations for 72 h A luminescent assay was used to assess cell proliferation. The broken lines indicate the theoretical additive effect, serving as a reference to determine the degree of synergy.

Since drug combinations are often employed in clinical settings for the treatment of immune-mediated diseases, we investigated the effects of the combination of physalin F and dexamethasone on concanavalin A-induced lymphocyte proliferation. Compared to the effects of each molecule alone, the combination of physalin F and dexamethasone reduced the EC_50_ values. When used individually, physalin F exhibited an EC_50_ of 0.65 µM, whereas, in combination with dexamethasone, the EC_50_ reduced to 0.006 µM. Similarly, dexamethasone alone presented an EC_50_ of 0.055 µM, which decreased to 0.004 µM when combined with physalin F. Indeed, the EC_50_ values of both drugs were reduced by at least 10-fold (Table 1). A combination index (CI) value of 0.17, along with a concave isobologram, revealed that physalin F and dexamethasone exhibit a synergistic effect (Table 1; Figure 4). The relevance of combined therapies in the treatment of immune-mediated diseases, such as multiple sclerosis, Crohn’s Disease, and psoriasis, has been previously demonstrated [32,33,34], and thus, physalin F may be an additional compound to be used in drug combination schemes.

Previous studies have already evaluated other biological activities of physalin F in combination with some reference drugs in other experimental settings. One study evaluated the anti-*T. cruzi* effect of physalin F with benznidazole, demonstrating that the combination reduced the number of infected macrophages and amastigotes per macrophage [15]. Furthermore, other combined therapy studies demonstrate that an extract rich in physalins B, D, F, and G showed anti-*T. cruzi* activity, when combined with benznidazole, against trypomastigotes, demonstrating a synergistic effect [35].

**Figure 5:**
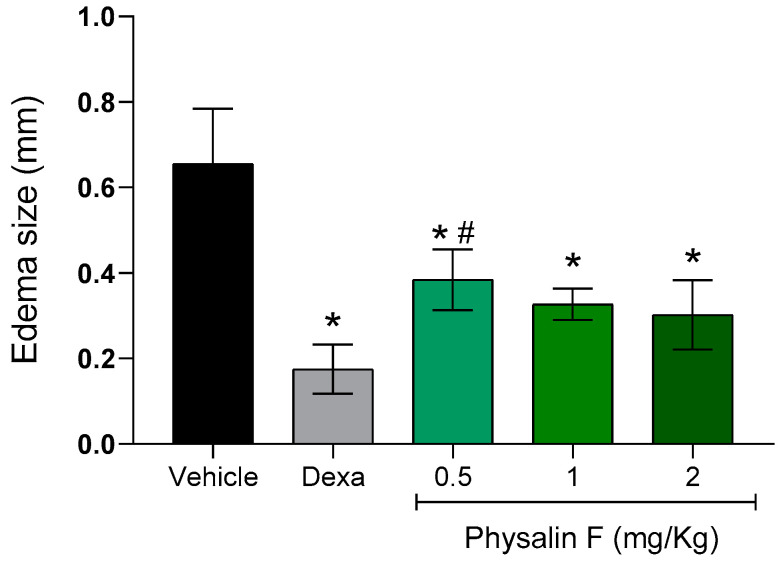
Physalin F reduces paw edema in a delayed-type hypersensitivity model. Male BALB/c mice (n = 6) were treated with physalin F (0.5, 1, and 2 mg/kg), dexamethasone (Dexa; 2 mg/kg), or vehicle (5% DMSO in saline) and challenged with 30 μL of a 2% heat-aggregated BSA suspension in saline, administered into the hind paw pad. Three hours after the challenge, the paw pad thickness was measured using a caliper, and the extent of swelling was calculated by subtracting the paw pad thickness before the challenge from that measured after the challenge. Values are expressed as mean ± S.E.M. of six mice per group, representing one of two independent experiments. Statistical significance was determined as * *p* < 0.05 compared to the vehicle group and # *p* < 0.05 compared to the dexamethasone group.

Lastly, the effect of physalin F was evaluated in a delayed-type hypersensitivity (DTH) model induced by bovine serum albumin (BSA) in BALB/c mice. On the seventh day after sensitization, groups of mice were treated intraperitoneally with different doses of physalin F (0.5, 1, and 2 mg/kg) or dexamethasone (2 mg/kg). Paw thickness measurements before and after the challenge were used as clinical indicators of hypersensitivity. As shown in Figure 5, treatment with physalin F at doses of 0.5, 1, and 2 mg/kg resulted in a reduction in paw edema. The 0.5 mg/kg dose reduced paw edema by more than 45% compared to the vehicle group; the group treated with 1 mg/kg achieved an approximately 52% reduction, while animals treated with the highest dose of physalin F (2 mg/kg) reduced paw edema by over 54% compared to the vehicle group. Treatment with dexamethasone (2 mg/kg) also significantly (*p* < 0.05) reduced paw edema compared to the vehicle group.

Few studies using animal models have focused on the effects of physalin F in lymphocyte-driven immune diseases. The delayed hypersensitivity assay, which primarily evaluates Th1 cell-mediated immunity, highlights the role of these cells as key producers of IFNγ [36]. The potent reduction in IFNγ levels by physalin F strongly supports these findings. Additionally, in two other in vivo models characterized by Th1-mediated responses, physalin F demonstrated similar effects: delaying allogeneic transplant rejection [21] and reducing inflammation in an arthritis model [37]. These results align with the findings from the DTH assay, reinforcing the immunomodulatory potential of physalin F.

## 3. Materials and Methods

### 3.1. Compounds

Physalin F was isolated from *Physalis angulata* L., as previously described [14]. Physalin F was initially dissolved in dimethyl sulfoxide (DMSO; PanReac, Barcelona, Spain) and subsequently diluted in Dulbecco’s modified eagle medium (DMEM; Life Technologies, Carlsbad, CA, USA) for in vitro assays. Dexamethasone and cyclosporin A (Sigma-Aldrich, St. Louis, MO, USA) were used as positive controls. The final concentration of DMSO was kept below 0.1% in all in vitro experiments. For in vivo assays, the compounds were solubilized in the saline solution containing 5% DMSO.

### 3.2. Animals

Male BALB/c mice (4–8 weeks old) were obtained from the animal facility of the Gonçalo Moniz Institute (Salvador, Brazil) and housed in sterilized cages under controlled environmental conditions (22 ± 2 °C, 55 ± 10% humidity), with ad libitum access to water and a balanced rodent diet. All experimental procedures were conducted in accordance with institutional animal ethics guidelines and approved by the animal ethics committee (Approval No. L-IGM-007/24).

### 3.3. Cytotoxicity to Mammalian Cells

To obtain the splenocytes, mice were euthanized, and an incision was made in the left hypochondrium to remove the spleen, which was then macerated with a syringe plunger. Then, splenocytes were centrifugated at 400× *g* for 10 min and subsequently resuspended in DMEM supplemented with 10% fetal bovine serum (FBS; GIBCO, Thermo Fisher Scientific, Waltham, MA, USA) and 1% penicillin–streptomycin (GIBCO). For cytotoxicity analysis, splenocytes were seeded in 24-well plates at a density of 1 × 10^6^ cells/well in DMEM medium supplemented with FBS and penicillin-streptomycin. Splenocytes were treated with physalin F (0.5, 1, or 2 µM) or dexamethasone (1 µM) for 72 h to assess compound cytotoxicity on mouse splenocytes. Following treatment, cells were centrifuged, washed twice with cold PBS, and stained with 2 µg/mL propidium iodide (Sigma-Aldrich) for 15 min in the dark. Samples were analyzed using a BD LSRFortessa SORP cytometer (Becton, Dickinson and Company; Franklin Lakes, NJ, USA), acquiring 10,000 events per sample. An excitation wavelength of 535 nm and an emission wavelength of 617 nm were used for PI fluorescence detection. Data were processed using FlowJo v.10 software (Tree Star, Ashland, OR, USA).

### 3.4. Lymphoproliferation Assay

Splenocytes obtained from BALB/c mice were plated in Greiner Black^®^ 96-well plates at a density of 1 × 10^6^ cells/well in DMEM supplemented with 10% FBS and 1% penicillin-streptomycin. Cells were either stimulated or not with concanavalin A (Con A—2 µg/mL; Sigma-Aldrich) and treated with physalin F (0.5, 1, or 2 µM) or dexamethasone (1 µM), followed by incubation for 72 h at 37 °C in a humidified atmosphere with 5% CO_2_. Following incubation, plates were centrifuged at 400× *g* for 10 min, the culture medium was removed, and 100 µL of CellTiter-Glo^®^ reagent (Promega, Madison, WI, USA) was added to each well, a luminescent marker of cell proliferation. Luminescence was measured using a microplate reader (Molecular Devices, San Jose, CA, USA).

### 3.5. Assessment of Cytokine Production

BALB/c mice splenocytes were seeded in 24-well plates at a density of 5 × 10^6^ cells/well in DMEM supplemented with 10% FBS and 1% penicillin–streptomycin. Cells were either stimulated or left unstimulated with concanavalin A (5 µg/mL) and treated with physalin F (0.5, 1, or 2 µM) or dexamethasone (1 µM) for 48 h. Following incubation, the cell-free supernatants were collected, and cytokine levels of IL-2, IL-4, IL-10, and IFN-γ were quantified using enzyme-linked immunosorbent assay (ELISA) DuoSet kits from R&D Systems (Minneapolis, MN, USA), following the manufacturer’s instructions. Data were acquired by colorimetric reading using a microplate reader (Molecular Devices) at a wavelength of 450 nm.

### 3.6. Calcineurin Activity

BALB/c mouse splenocytes were seeded in 24-well plates at a density of 1 × 10^6^ cells/well in DMEM supplemented with 10% FBS and 1% penicillin–streptomycin. Cells were either stimulated or left unstimulated with concanavalin A (5 µg/mL) and treated with physalin F (1 µM) or cyclosporin A (1 µM) for 48 h. Following treatment, cells were lysed in a buffer containing protease inhibitors, centrifuged at 10,000× *g*, and the supernatant was collected for analysis. Equal amounts of protein (5 µg per sample) were used for the calcineurin activity assay, performed using the Calcineurin Cellular Activity Assay Kit (Enzo Life Sciences, Farmingdale, NY, USA). Colorimetric measurements were conducted at 620 nm, and the amount of phosphate released by calcineurin was quantified using a standard calibration curve.

### 3.7. Combination Therapy

For in vitro drug combination analysis, two-fold serial dilutions of each drug (physalin F and dexamethasone), tested individually or in fixed combinations, were added to splenocyte cultures (1 × 10^6^ cells per well) in Greiner Black^®^ 96-well plates. Cells were incubated for 72 h with or without concanavalin A (2 µg/mL) stimulation. For the isolated compounds, serial dilutions were made from 2 µM and 0.5 µM of physalin F and dexamethasone, respectively, with a total of 8 concentrations in a ratio of 1:2. Serial double dilutions were performed in triplicate in ratios of 1:1, 2:1, and 1:2, dexamethasone and physalin F, respectively, using BALB/c mice splenocytes. For each proportion, an EC_50_ value was calculated for each drug and combination. The fractional inhibitory concentrations (FIC) were calculated by [EC_50_ when combined/EC_50_ isolated drug]. After 72 h of treatment, the plate was centrifuged for 10 min at 400× *g*. The culture medium was removed, and 100 µL of CellTiter Glo^®^ reagent was added. Luminescence was measured using a microplate reader (Molecular Devices). Drug interactions were evaluated by calculating the combination index (CI) using the Chou–Talalay method [38] and constructing isobolograms via the fixed-ratio method, as previously described [39].

### 3.8. Delayed-Type Hypersensitivity Assay

Male BALB/c mice (8–12 weeks of age) were sensitized by subcutaneous injection of 50 µg crystallized bovine serum albumin (BSA; Sigma-Aldrich) emulsified in 20 µL of Complete Freund’s Adjuvant (CFA; Sigma-Aldrich) at the base of the tail. After seven days, animals were randomly allocated into six groups (6 mice per group, total of 36 animals for experiment) and received intraperitoneal injections of physalin F (0.5, 1, or 2 mg/kg) or dexamethasone (2 mg/kg) or vehicle (10% DMSO in saline). Treatments were administered 24 and 3 h prior to challenge. The delayed-type hypersensitivity (DTH) response was elicited by subcutaneous injection of 30 µL of a heat-aggregated 2% BSA suspension in saline into the plantar pad, following a previously established protocol [40]. Paw thickness was measured using calipers before and 3 h after the challenge, and swelling was determined by calculating the difference between pre- and post-challenge measurements.

### 3.9. Statistical Analysis

The significance of differences between groups was evaluated using one-way ANOVA, followed by the Newman–Keuls multiple-comparison post-test. Analyses were performed using GraphPad Prism version 8.0 (GraphPad Software, San Diego, CA, USA). Results were considered statistically significant when *p*-values were less than 0.05 (*p* < 0.05).

## 4. Conclusions

The results highlight physalin F as a compound with potent immunosuppressive activity on activated lymphocytes. The findings suggest that physalin F primarily exerts its immunosuppressive effects by inhibiting the calcineurin pathway. Furthermore, it demonstrates synergistic activity when combined with dexamethasone, enhancing its therapeutic potential in immune-mediated diseases. Notably, this study is the first to demonstrate the immunosuppressive effect of physalin F in an experimental model of delayed-type hypersensitivity, further underscoring its potential in the treatment of Th1-mediated diseases.

## Figures and Tables

**Table 1:** Concentration reductions and combination indexes for immunosuppression by physalin F and dexamethasone.

Compounds	EC_50_ ± S.D (μM) ^a^		
	Drug Alone	Combination	CI ^b^
Physalin F	0.65 ± 0.1	0.006 ± 0.007	0.17 ± 0.06
Dexamethasone	0.055 ± 0.01	0.004 ± 0.003	

^a^ EC_50_ values were determined from quadruplicate concentration measurements across two independent experiments. ^b^ Combination index (CI). Cutoff: CI value of 0.1–0.7, synergism; 0.7–0.85, moderate synergism; 0.85–0.9, slight synergism; 0.9–1.1, additivity; >1.1, antagonism. S.D. = standard deviation.

## Data Availability

Data are contained within the article and Supplementary Materials.

## Supplementary Materials

The following supporting information can be downloaded at: https://www.mdpi.com/article/10.3390/molecules30040916/s1, Figure S1: Physalin F did not show cytotoxicity at the tested concentrations. The untreated and non-stimulated control is shown in (A), while dexamethasone at 1 µM is in (B). Physalin F at concentrations of 0.5 µM, 1 µM, and 2 µM is shown in (C), (D), and (E), respectively.

## Abbreviations

BSA: bovine serum albumin; CFA: complete Freund’s adjuvant; CI: combination index; Con A: concanavalin A; CsA: cyclosporin A; Dexa: dexamethasone; DMEM: Dulbecco’s modified eagle medium; DMSO: dimethyl sulfoxide; EC_50_: effective concentration 50%; FBS: fetal bovine serum; HAM/TSP: HTLV-1-associated myelopathy/tropical spastic paraparesis; HTLV-1: human T-lymphotropic virus type 1; IFN-γ: interferon gamma; IL: interleukin; LC_50_: lethal concentration 50%; NFAT: nuclear factor of activated T-cells; NF-κB: nuclear factor kappa B; PBMC: peripheral blood mononuclear cells; Phy. F: physalin F; PI: propidium iodide; TNF-α: tumor necrosis factor alpha.
