# Supplementary material for: Physalin F, a Potent Inhibitor of Lymphocyte Function, Is a Calcineurin Inhibitor and Has Synergistic Effect with Dexamethasone
Source: Molecules. 2025 Feb 16;30(4):916. doi: 10.3390/molecules30040916 (PMC11858416; doi:10.3390/molecules30040916)
Supplement: Supplementary file 1 [file molecules-30-00916-s001.zip › molecules-3436856-supplementary.pdf]

Supplementary Information

Physalin F, a potent inhibitor of lymphocyte function, is a cal-cineurin inhibitor and has synergistic effect with dexame-thasone

Dahara Keyse Carvalho Silva [1], Laura Beatriz da Cruz Novo [1], Ivone Maria Ribeiro [2], Breno Cardim Barreto [3], Luiza Carolina França Opretzka [3], Cássio Santana Meira [1,3] and Milena Botelho Pereira Soares [1,3*]

[1] Gonçalo Moniz Institute, Oswaldo Cruz Foundation (FIOCRUZ), 40296-710 Salvador, Bahia, Brazil.

[2] Laboratory of Natural Products Chemistry - PN2, Farmanguinhos, Oswaldo Cruz Foundation (FIOCRUZ), Rio de Janeiro 22.775-903, RJ, Brazil.

[3] Institute for Innovation in Advanced Health Systems, SENAI CIMATEC, Salvador 41650-010, BA, Brazil.

Supplementary Information

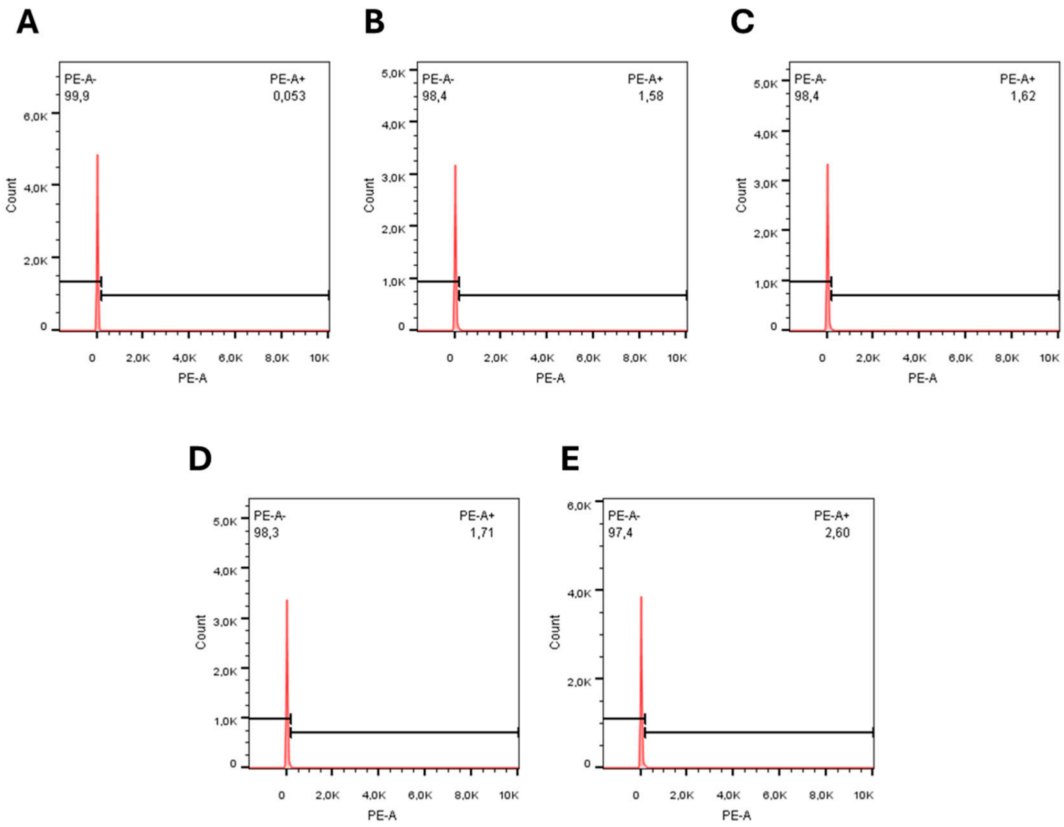

Figure S1. Physalin F did not show cytotoxicity at the tested concentrations. The untreated and non-stimulated control is shown in (A), while dexamethasone at 1 µM is in (B). Physalin F at concentrations of 0.5 µM, 1 µM, and 2 µM is shown in (C), (D), and (E), respectively.
